# Oral Healthcare during Pregnancy: Its Importance and Challenges in Lower-Middle-Income Countries (LMICs)

**DOI:** 10.3390/ijerph191710681

**Published:** 2022-08-27

**Authors:** Shah Saif Jahan, Ehsanul Hoque Apu, Zeeba Zahra Sultana, Md Irteja Islam, Nazeeba Siddika

**Affiliations:** 1 School of Allied Health, Faculty of Health, Education, Medicine and Social Care, Anglia Ruskin University, Chelmsford CM1 1SQ, UK; 2 Centre for International Public Health and Environmental Research, Bangladesh (CIPHER,B), Dhaka 1207, Bangladesh; 3 Department of Biomedical Engineering, Institute of Quantitative Health Science and Engineering, Michigan State University, East Lansing, MI 48824, USA; 4 Division of Hematology and Oncology, Department of Internal Medicine, Michigan Medicine, University of Michigan, Ann Arbor, MI 48105, USA; 5 Global Health Institute, North South University, Dhaka 1229, Bangladesh; 6 Department of Public Health, North South University, Dhaka 1229, Bangladesh; 7 Sydney School of Public Health, Faculty of Medicine and Health, The University of Sydney, Sydney, NSW 2006, Australia; 8 Centre for Health Research and Faculty of Health, Engineering and Sciences, The University of Southern Queensland, Toowoomba, QLD 4350, Australia; 9 Department of Epidemiology and Biostatistics, College of Human Medicine, Michigan State University, East Lansing, MI 48824, USA

Oral health is essential in general health and well-being to maintain overall quality of life. Pregnant women are more likely to develop gingivitis, an early stage of periodontal disease, which occurs when the gums become red and swollen from inflammation that could be aggravated by hormonal changes during pregnancy [1]. This Special Issue, entitled “Oral health and manifestations related to adverse pregnancy outcomes, and their preventive measures”, aims to find the true association between oral manifestations and adverse pregnancy outcomes, the factors that modify this effect, the barriers to accessing dental care for pregnant women, and the impact of potential interventions to treat or reduce adverse pregnancy outcomes while ensuring safe health for both mother and fetus.

Pregnancy is a natural physiological process accompanied by temporary changes in women’s physical structure, hormone levels, metabolism, and immune systems [2,3]. An increased carbohydrate consumption, vomiting acid, reduced saliva production, and an increased acidity of saliva are associated with pregnancy; however, this may alter the risk of oral diseases, such as periodontal disease and dental caries [4,5]. Changing oestrogen levels and progesterone makes the mouth absorbent and the host’s immune system less efficient, increasing the chance of a dental infection [6]. It has been observed that dental caries and gingivitis occurred 1.97 times and 1.81 times more frequently in pregnant women than in non-pregnant women, respectively [7].

Gingivitis and periodontal diseases are associated with adverse pregnancy outcomes, including preterm birth (PTB) and low birth weight (LBW) [8]. Since 1996, numerous studies have highlighted the connection between dental health and undesirable pregnancy outcomes, when periodontal disease was first reported as a potential risk factor for premature delivery (PTD) [8,9]. PTB rates were shown to rise in tandem with the severity of gingivitis and periodontitis. Ref. [10] Similarly, periodontitis was also shown to be substantially linked to LBW/PTB new-borns [11]. These outcomes included uterine leiomyoma, gestational hypertension, PTB, PTD, and small gestational age [12]. Moreover, in women who have a lot of cavity-causing bacteria during pregnancy, after delivery, these bacteria could transmit from their mouth to the mouth of their baby [13]. Early contact with these bacteria and other sugar sources, through frequent snacking or taking a bottle to bed, can lead to early childhood cavities and the need for extensive dental care at a young age [14]. Considering all of these factors, it is evident that ensuring dental care during pregnancy is very important to considerably improve mothers’ oral health and lower the chance of babies having early dental caries.

Dental caries and periodontal disease among pregnant women are largely preventable. Yet, advancements in oral health and access to oral healthcare face many challenges, particularly in lower-middle-income countries (LMICs) [15,16]. Access to oral healthcare varies from 35% in low-income countries and 75% in upper-middle income countries [17]. In the United States, only 46% of pregnant women have oral prophylaxis (dental cleaning) during pregnancy, and this figure is much lower for socially disadvantaged women [18]. To some extent, there are regular dental coverage programs available for pregnant women in high-income countries. However, there is no routine dental check-up recommended during pregnancy in most LMICs [19]. The most significant impediments are a lack of information for pregnant women regarding the importance for dental appointments during pregnancy, misconceptions regarding the safety of dental treatment during this time, and the lack of a perceived need to attend the dentist during pregnancy [20,21]. Access to dental care is also related to income level; women with low incomes are less likely to receive dental care before and during pregnancy than those with higher incomes [22]. Additionally, in LMICs, there is a lack of affordable and institutional childcare services. As a result, when pregnant women need dental care, they often have trouble finding someone to babysit their older children [23].

In many countries, dentists and midwives also acknowledged a lack of understanding regarding prenatal dental healthcare and asked for further training in this field. Although current standards indicate the safety of dental treatments in all trimesters of pregnancy, many prenatal care professionals were unclear about the safety of dental procedures during pregnancy and unwilling to treat pregnant women [24].

The World Health Organization has placed a greater focus on the idea of an integrated health service delivery to deal with limited resources, especially a lack of human resources in LMICs [25]. For this integration to work, general healthcare providers and dentists need to work together. Even though there has been some progress, there are still problems, which makes this health system far from perfect [26]. If health centre professionals do not work together, dentists may not want to treat pregnant women. Oral screenings for pregnant women are often not performed due to the medical professionals’ beliefs that screenings and referrals for dental care during pregnancy are not their primary responsibilities [27].

Another factor is physiological and psychological changes during pregnancy [19,21,26]. Pregnant women often believe that physical changes and changes in their mental health make it hard for them to access dental care, and it is difficult to change this tendency. Receiving dental treatment from male dentists is unpleasant due to the religious implications of dentists of different genders providing care, which may explain why some women are reluctant to receive dental treatments from male dentists [28]. However, some women prefer male dentists, assuming that they had more experience and better skill sets than female dentists. Despite female dentists working at health facilities, other health professionals are not willing to have patients referred to them [24,27]. Understanding the reasons that pregnant women do not seek dental care during this sensitive period is important for creating future programmes to improve and maintain oral health for this vulnerable group of people.

This editorial addresses the importance of oral healthcare during pregnancy and the obstacles that prevent pregnant women from using dental services. There is a substantial lack of current knowledge on this topic due to the availability of only a limited number of studies on that aim to understand the complete context of this issue and to plan for effective interventions. More studies from LMICs are needed because of a lack of knowledge, the limited availability and accessibility of public dental services, the high cost of private dental care, and the absence of interprofessional collaboration and cultural taboos. The implementation of effective oral health promotional initiatives for pregnant women requires extensive research to identify the gaps in the knowledge.

We hope that the readers of the *International Journal of Environmental Research and Public Health* will discover the true association between oral manifestations and adverse pregnancy outcomes, effect modifying factors, and barriers to accessing dental care for pregnant women, as well as the strategies to implement potential interventions that improve the oral health of pregnant women, while reducing associated adverse pregnancy outcomes in the future.
